# Computational Modeling of Ganglion Cell Bicolor Opponent Receptive Fields and FPGA Adaptation for Parallel Arrays

**DOI:** 10.3390/biomimetics9090526

**Published:** 2024-08-31

**Authors:** Hui Wei, Wenbo Yao

**Affiliations:** Laboratory of Algorithms for Cognitive Models, School of Computer Science, Fudan University, Shanghai 200438, China; 22212010047@m.fudan.edu.cn

**Keywords:** brain-like model, retina, programmable devices, receptive fields

## Abstract

The biological system is not a perfect system, but it is a relatively complete system. It is difficult to realize the lower power consumption and high parallelism that characterize biological systems if lower-level information pathways are ignored. In this paper, we focus on the K, M and P pathways of visual signal processing from the retina to the lateral geniculate nucleus (LGN). We model the visual system at a fine-grained level to ensure efficient information transmission while minimizing energy use. We also implement a circuit-level distributed parallel computing model on FPGAs. The results show that we are able to transfer information with low energy consumption and high parallelism. The Artix-7 family of xc7a200tsbv484-1 FPGAs can reach a maximum frequency of 200 MHz and a maximum parallelism of 600, and a single receptive field model consumes only 0.142 W of power. This can be useful for building assistive vision systems for small and light devices.

## 1. Introduction

Primates rely extensively on their visual systems to navigate their environment. Evolved through natural selection, these systems are highly robust and efficient, becoming a major focus in brain-inspired computing research. Although modern deep learning networks are effective, they demand significant computational resources and operate on Von Neumann (VN) architecture-based machines. Despite accelerated computing techniques like GPUs, these models lack the spatiotemporal parallelism found in biological neural systems (BNNs). This parallelism occurs across multiple scales, including the nucleolus, loop, cellular, synapse, molecular and electrochemical reaction levels [1].

Unlike conventional computing paradigms with centralized topology and input–output constraints, BNNs feature decentralized processing with balanced loads. This study leverages the FPGAs to emulate the spatiotemporal parallelism of BNNs. With their multiple logic units, these platforms offer a means for simulating and optimizing signal flow in biologically inspired parallel computing models.

Leveraging the programmable hardware architecture of Field-Programmable Gate Arrays (FPGAs), this study aims to emulate the spatial and temporal parallel characteristics of biological neural networks. By exploiting multiple logic units that can simultaneously execute diverse tasks, FPGA technology offers a promising way to simulate and optimize signal flow. This approach helps in realizing a truly biologically inspired parallel mechanism.

Moore’s Law, traditionally driven by transistor miniaturization, faces manufacturing limitations. Pursuing true spatiotemporal parallelism demands a departure from the VN model, which segregates computation and storage, imposing inherent constraints.

Despite advancements in computational speed through technologies such as GPUs and multi-core systems, the serial nature of processing remains the fundamental bottleneck. The separation of storage and computation incurs energy expenditure on data pathways and operational overheads, including instruction retrieval and execution.

Conversely, the human brain executes complex reasoning, decision-making and autonomous functions, consuming approximately 20 W of energy. In comparison, the average computer needs approximately 250 W to recognize 1000 objects [2]. This contrast highlights the structural disparity between the human brain and VN computers.

Some brain-inspired chips enhance parallel computation by increasing CPU-centric cores for deep learning, while others mimic the biological nervous systems [3]. The former retains the VN architecture, and the latter explores analog or digital designs. However, analog chips incline towards error accumulation and manufacturing constraints, rendering digital-electric brain-inspired chips a practical option.

**Figure 1:**
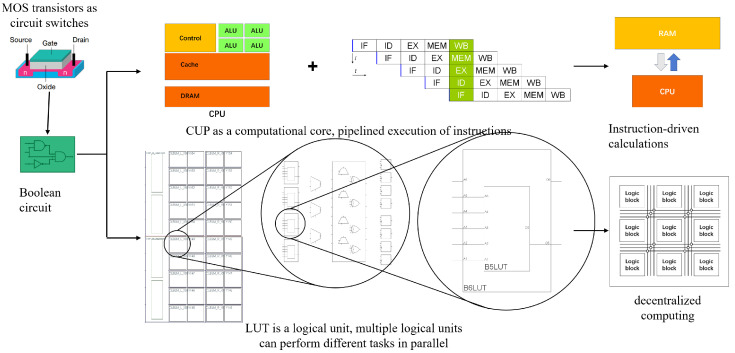
Comparative analysis of VN-style computers and FPGA.

Among digital-electric platforms, field programmable analog arrays (FPAAs) provide large bandwidths but suffer from limited parameter adjustability and high costs [4,5]. However, field programmable gate arrays (FPGAs) provide a low-power, high-performance hardware programmable solution [6,7,8] which has increased its use in neural engineering [9]. Consequently, this paper uses FPGA for parallel computational modeling of adaptive ganglion cell two-color opponents. Figure 1 compares VN computers with these reconfigurable devices (FPGAs). While both utilize MOS transistors and Boolean circuits, these devices use look-up tables (LUTs) for hardware programmability. The extensive utilization of LUTs allows for efficient retinal encoding space-time parallelism.

Based on digital circuit principles, these devices operate by encoding circuit logic into a truth table, which is used to perform computations at runtime. This design eliminates the need for dedicated computation components. The hardware device uses circuit logic to represent the entire computational model. Its configurations are stored directly in hardware. This allows parallel information transfer between circuits, similar to impulse signal transmission in living organisms. This feature supports spatiotemporal processing, rendering these devices ideal for modeling the retina’s parallel encoding.

Moreover, FPGA configurations are directly stored in hardware. The parallel transfer of information between circuits mirrors the parallel transmission of impulse signals observed in living organisms. This characteristic allows processing to happen simultaneously in both time and space. As a result, FPGAs are well-suited for modeling pathways. These pathways show real parallelism, similar to the encoding process in the biological retina.

**Figure 2:**
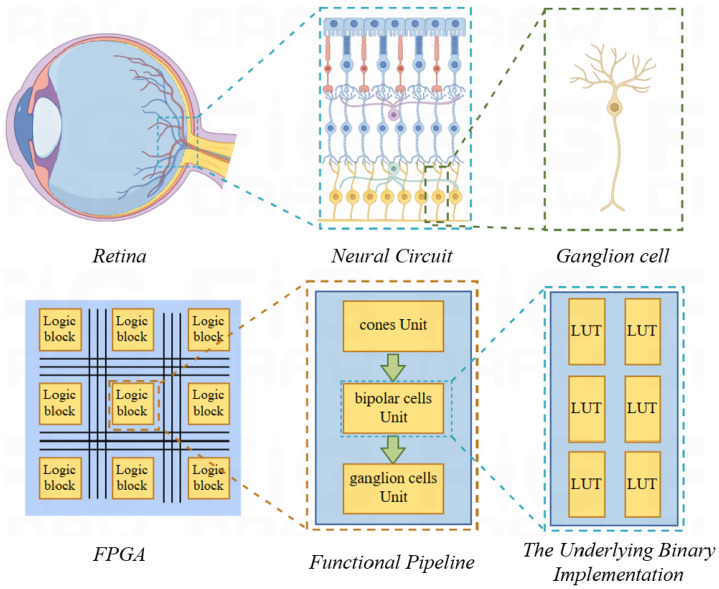
FPGA design for retinal channel.

Figure 2 exhibits the retina’s complex network with high parallelism. FPGAs, with their parallel architecture, can efficiently map the retina’s parallel processing structure. The retina is decomposed into independent units. Each unit can be implemented with a few LUTs. This allows a multitude of parallel retinal functions to be realized on a single FPGA.

## 2. Related Works

Research on brain-inspired computing often focuses on the hierarchical structure of the cerebral cortex and neural synapses [10]. Deep learning constructs hierarchical models by layering various features within the input data. These frameworks use integrated transistors for Boolean logic but maintain distinct processing and storage units. This distinction contrasts sharply with biological systems, where computation and storage are integrated within parallel pathways.

In the primate retina, photoreceptor cells convert light signals into bioelectrical signals [11,12,13]. These signals traverse the retina and the LGN before reaching the cerebral cortex. The complex neural pathways, though not fully understood, operate simultaneously, with each pathway serving a specific function [14]. At the retinal level, optic cone cells are crucial for color vision, requiring specific luminance conditions [15,16,17]. At the origin of color vision, signals must undergo initial color processing before cortical transmission.

This paper focuses on the retinal M, P and K visual pathways, extending to the LGN. These pathways support small, high-precision vision (M), large, low-precision (P) and chromatic signal modulation (K) [18]. By examining these pathways, the paper elucidates visual perception and processing in biological systems.

Numerous studies have analyzed and modeled retinal microcircuits onFPGAs [13,19,20]. Further research has simulated retinal functions on FPGAs using cameras as inputs [21,22]. While these detailed models are commendable, this paper takes a broader approach, focusing on parallel pathways from optic cones in the retina to the LGN.

This paper primarily relies on biological mechanisms [18], building the M, P and K visual pathways from the retina to the LGN. It also explores a single receptive field implementation and maximum parallel receptive fields on various FPGAs. This endeavor seeks to underscore the complexity and efficiency of biological systems from a novel vantage point.

## 3. Biological Basis of Parallel Pathways in the Primate Retina and Their Computational Modeling

### 3.1. Mechanisms of Primate Retinal Coding

**Figure 3:**
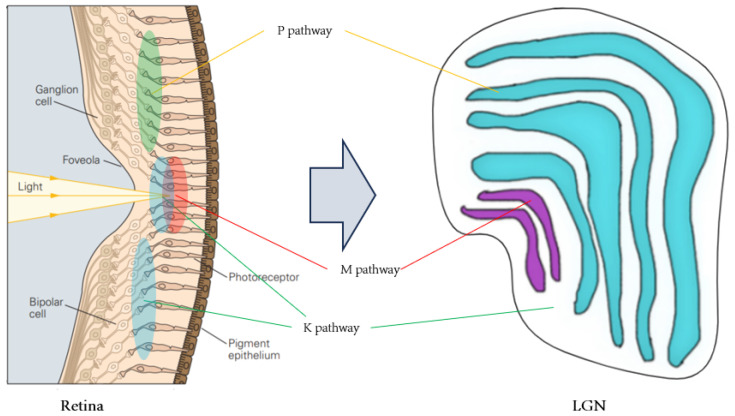
Visual information transfer from the retina to the LGN.

Color vision, originating in the retina, has been a neuroscientific focus. The retina contains nearly 100 neuronal types [23], with complex structures and interrelationships under investigation. Optic cones and rod cells are the main signal-receiving units, with rod cells responsible for black-and-white vision in low light. Under normal light, the photoreceptor cell layer contains L-type, M-type and S-type cone cells, with peak spectral absorptions at 440 nm (blue), 535 nm (green) and 565 nm (red), respectively. The parallel pathways encoded in the retina begin with these cone cells, and the transfer of visual information from the retina to the LGN is studied through horizontal cells (Figure 3).

This paper focuses on the visual parallel pathways between the retina and the LGN, which includes the P pathway (blue-yellow opponent), the M pathway (red-green opponent) and the K pathway (luminance opponent). The LGN is divided into six main layers: the dorsal small-cell P layers (layers 3, 4, 5 and 6), the ventral large-cell M layers (layers 1 and 2) and the interleaved K-cell layers. The similarities and differences among the K, M and P pathways are depicted in Table 1:

#### 3.1.1. K-Pathways and Their Computational Models

**Figure 4:**
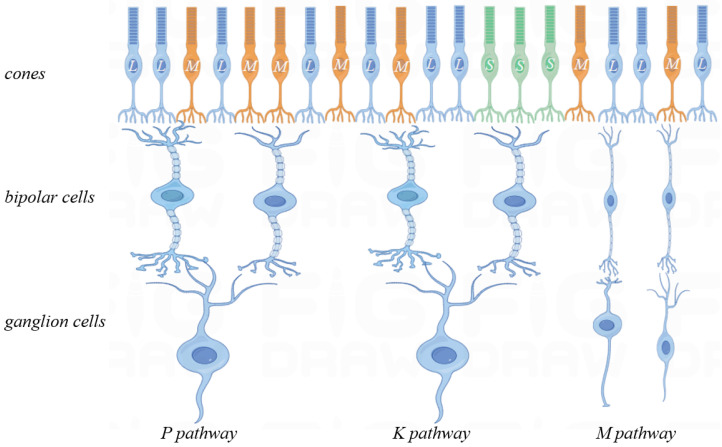
Neural channels of the P, K and M pathway.

Blue, a short wavelength, mainly activates S-type cone cells in the retina. L-type cone cells absorb long wavelengths, while M-type cone cells capture medium wavelengths, corresponding to the three primary colors. Yellow absorption results from a mix of L- and M-type cells [18]. Figure 4 depicts the blue-yellow opponent pathway, where horizontal cells connect L, M and S cone cells with ganglion cells, which then transmit signals to the K-cell layer of the LGN.

Among the K, M and P channels, only K and M display bicolor opponents, with peripheral inhibitory and central excitatory signals from distinct cone types. The P pathway, conversely, uses both L and M cones, constituting a homochromatic opponent system. Despite the similarity, both pathways still exhibit opponent interactions, involving cones of the same type.

**Figure 5:**
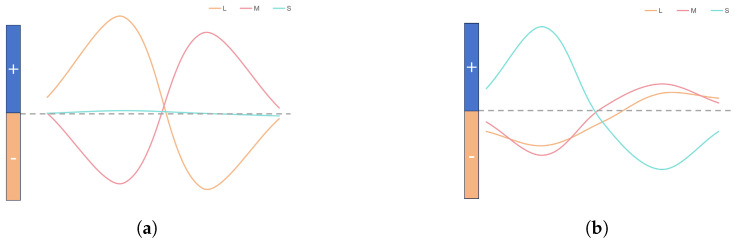
Red-green and blue-yellow opponent interactions. (**a**) Red-green opponent, L and M cones are opposed. (**b**) Blue-yellow opponent, S cones oppose a combined signal from L and M cones.

Figure 5 illustrates blue-yellow opponent interactions. The M pathway’s receptive field shows red-green opposition, with minimal S-cone involvement. The L and M cones generate both positive and negative signals, resulting in combined effects. Consequently, the three cones exhibit opponent effects. The K pathway has S cones centrally located and L and M cones peripherally, while the M pathway features a blue center and yellow periphery. Moreover, L and M cone cells superimpose to form a periphery equivalent to the yellow color [24].

In this study, we employ Rodieck’s Gaussian difference model [25] to depict the center opponent. Convolution extracts center features, followed by red and green feature extraction. These features are combined to form a yellow signal, contrasting with a blue center (Figure 5).

(1) $$g\left(x,y,\sigma \right)=1/\left(2\pi \sigma^{2}\right)e^{-\left(x^{2}+y^{2}\right)/2\sigma^{2}}$$

(2) $$g_{1}\left(x,y,\sigma_{1}\right)=1/\left(2\pi \sigma_{1}^{2}\right)e^{\left(x^{2}+y^{2}\right)/2\sigma_{1}^{2}}$$

*g* denotes the image information in the receptive field, and *g*_1_ denotes the weight information of the receptive field, where σ and $\sigma_{1}$ are the size of the receptive field for *g* and $g_{1}$, respectively; *x*, *y* denote the positional relationship of the center of the receptive field. Since the size of the receptive field has been determined in biology, the output of the ganglion cells can be simplified:

(3) $$O\left(x,y\right)=g\left(x,y\right)*g_{1}\left(x,y\right)$$

where * represents convolution operation, *O(x,y)* signifies the computational form of *L(x,y)*, *M(x,y)*, *S(x,y)*. K-pathway opponent computation prioritizes blue-yellow differentiation:

(4) $$C_{B-Y}=S_{cen}-\left(M_{per}+L_{per}\right)/2$$

where $C_{B-Y}$ indicates the opponent output; $S_{cen}$ denotes the S-shaped cone response in the central region; $M_{per}$ and $L_{per}$ represent the cone responses in the peripheral region.

#### 3.1.2. M-Channels and Their Computational Models

The M-pathway utilizes L and M cone cells to process visual data. Confined to the central fovea, it comprises a single midget ganglion cell linked to a solitary midget bipolar cell. This bipolar cell connects to one L or M cone, relaying signals to the LGN’s M-cell layer. L-M cone opposition occurs within the central fovea’s bipolar cells (Figure 5). Beyond the fovea, L and M collaborate for luminance encoding [18]. The fovea enhances image acuity, while the periphery sacrifices detail for energy efficiency. Figure 4 depicts the M-channel’s red-green opposition. The red center responds to red stimuli, while the green periphery detects green. S cones are excluded from this process. We model this red-green opposition using convolution to extract red center and green periphery features, demonstrating their mutual antagonism (Figure 5). The opponent relationship is calculated as follows:

(5) $$C_{R+G}=\left(L_{cen}-L_{per}\right)/2+\left(M_{cen}-M_{per}\right)/2$$

where $C_{R+G}$ depicts the M-pathway opponent output, $L_{cen}$, $M_{cen}$, $L_{per}$ and $M_{per}$ signify L and M cone responses in center and peripheral regions, respectively.

#### 3.1.3. P-Pathways and Their Computational Models

The P pathway excludes S cones, focusing on L and M cone light signal conversion. These cone cells convert light signals into bioelectrical signals, relaying them to non-opponent bipolar cells. They transmit visual signals to P-cell LGN neurons. Figure 4 depicts diffuse optic cone bipolar cells connecting to L and M cones, similar to H1 cells, without S-cone input. Bipolar cells exhibit non-opponent center-peripheral receptive fields before signaling to ganglion cells. L and M cone proportionality, along with H1 cells, inhibits the peripheral region within diffuse bipolar cells.

Bipolar cell actions are consistent across types, rendering separate models redundant. Consequently, the S cone is excluded from opponent considerations. The L and M cones exhibit monochromatic, not two-color opposition, with both the center and periphery receiving L and M cone signals. Their opponent relationship is computed as follows:

(6) $$C_{R-G}=\left(L_{cen}-M_{per}\right)/2$$

where $C_{R-G}$ indicates the P-pathway opponent output, with $L_{cen}$ representing L and M cone responses in center and periphery.

### 3.2. M-Pathway and K-Pathway Receptive Field Sizes

Evolution prioritizes sufficiency over perfection. Human vision selectively processes information via a focused attention mechanism, optimizing energy use. This mechanism channels critical information through the central fovea’s M pathway, while peripheral details are transmitted through the P pathway. The K supports both channels by providing color context.

The human retina’s central concavity represents exceptional nerve cell density, providing high visual resolution. This area transmits the most critical visual data, despite its limited perspective.

**Figure 6:**
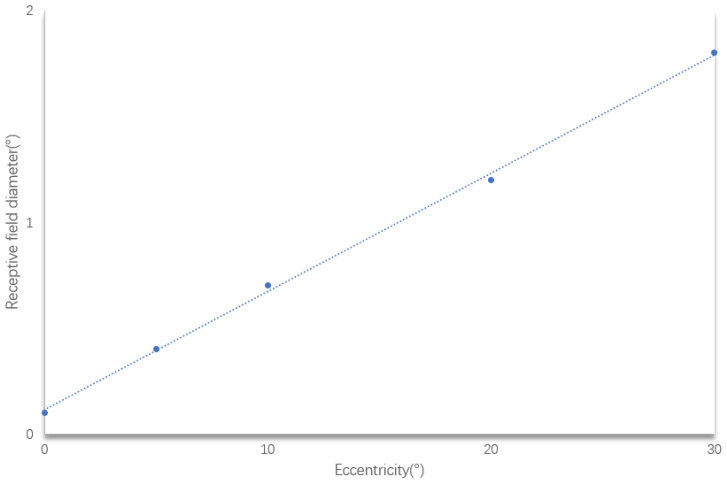
Receptive field range as a function of eccentricity.

The term ”eccentricity” [26] measures the distance from the central concavity, and it demonstrates receptive field increasentricity (Figure 6).

### 3.3. Parallel Computational Modeling of Retinal Coding

**Figure 7:**
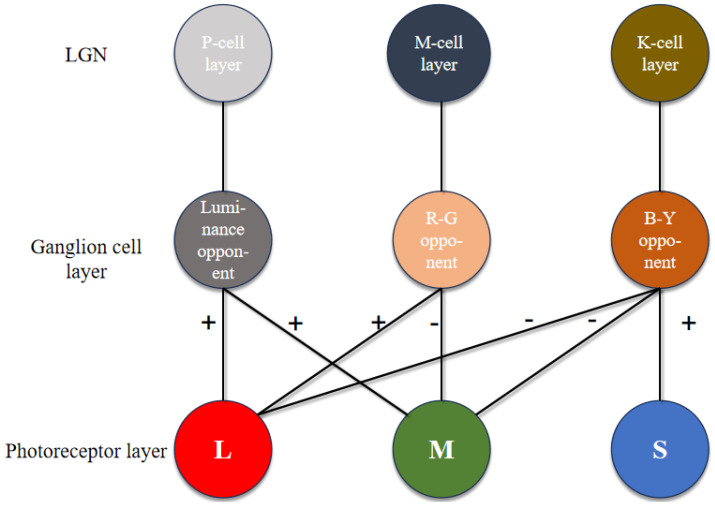
Coding calculation model.

Figure 7 presents the retinal coding model. L, M and S represent cone cells. P exhibits luminance opponent, while L and M cones show no opposition. R-G opponent defines the M channel with a red-centered green periphery, while B-Y opponent represents the K-cell layer.

Ganglion cells’ opponent computations require convolution-like operations within the receptive field. FPGAs, lacking CPU-inspired computational cores, cannot rely solely on loops for convolution. In contrast, per-receptive-field computations enable parallel processing.

The K, M and P channels are considered spatiotemporal independent parallel structures, intersecting only at the retinal receptor layer. Each channel transmits signals via ganglion cells to the LGN, with each cell possessing a receptive field for parallel signal transmission. This transmission represents parallel receptive field computation. While biological systems lack distinct channel regions, we compute K, M and P channels in parallel on a receptive field basis within a specific time frame.

## 4. Ganglionic Two-Color Opponent FPGA Adaptation Implementation

**Figure 8:**
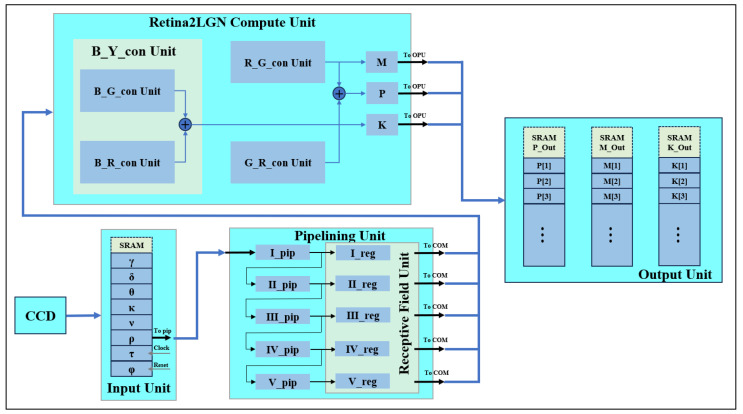
Computational model based on primate retinal coding channels.

Three parallel channels (luminance, red-green and blue-yellow) generate independent outputs. The R, G and B register pipeline simulates the L, M and S cone photoreceptor layers. A parallel computing module represents three visual pathways, producing corresponding images. We implement the three modules to generate pathways (Figure 8) and simulate K, M and P channels, each representing a GC-LGN component. While computations differ, all channels show opponent properties.

The human eye captures light signals, converting them into bioelectrical signals via photoreceptor cells. These signals are transmitted through visual channels to the LGN, ultimately reaching the cerebral cortex for image formation. Photoreceptors include L, M and S types cone cells and rod cells. These receptors comprise rods for low-light, black-and-white vision and cones for color vision under brighter conditions. L, M and S-type cone cells are sensitive to long, medium and short wavelengths.

This study encompasses three register matrices that simulate parallel L, M and S cone outputs. To mimic the retinal signal reception, line cache pipelines are used. While full parallel image transmission is resource-intensive, biological vision’s complexity cannot be fully captured by a single-feature model.

FPGA register matrices to simulate parallel retinal signal transmission to higher levels. These matrices represent receptive fields, enabling parallel three-channel computation. Luminance, red-green and blue-yellow opponent channels are implemented in parallel. An R, G and B register pipeline constructs the L, M and S cone photoreceptor layers. The parallel computing module represents three visual pathways, generating corresponding images.

FPGAs are employed to implement the K, M and P channel image generation. Figure 8 shows the model simulating biological parallel pathways, with each module replicating a GC-LGN (ganglion cell-lateral geniculate nucleus) pathway segment. These pathways, though computed differently, share opponent characteristics.

FPGA registers enable parallel information transfer, mimicking biological receptive field transmission. Register matrices model receptive field and facilitate parallel three-channel computation.

### 4.1. FPGA Design for Parallel Delivery of L, M, S Cone Cells in the Receptive Field

**Figure 9:**
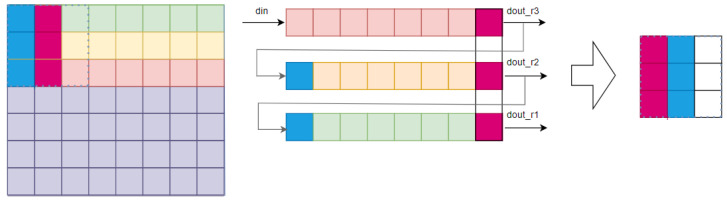
Optimized parallel processing of image information.

Primate vision relies on K, M and P channels between the GC and LGN. FPGAs read images sequentially, but a pipeline stores data in a 3 × 3 sensory field to restore spatiotemporal parallelism (Figure 9).

**Figure 10:**
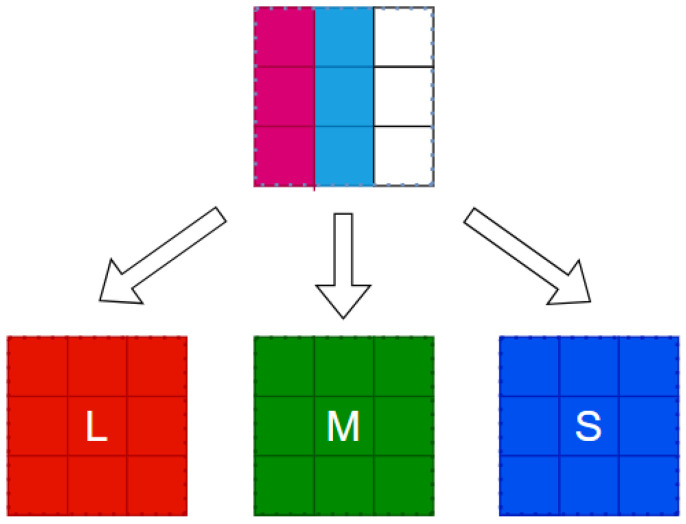
Extraction of L, M and S sensory field information in FPGAs.

To enable parallel receptive field processing, the pipeline’s length matches the image width. Receptive field depth determines the pipeline’s cache line count (Figure 9). A second register pipeline, matching receptive field dimensions, stores previous image data, resulting in complete parallel receptive field processing (Figure 10).

**Figure 11:**
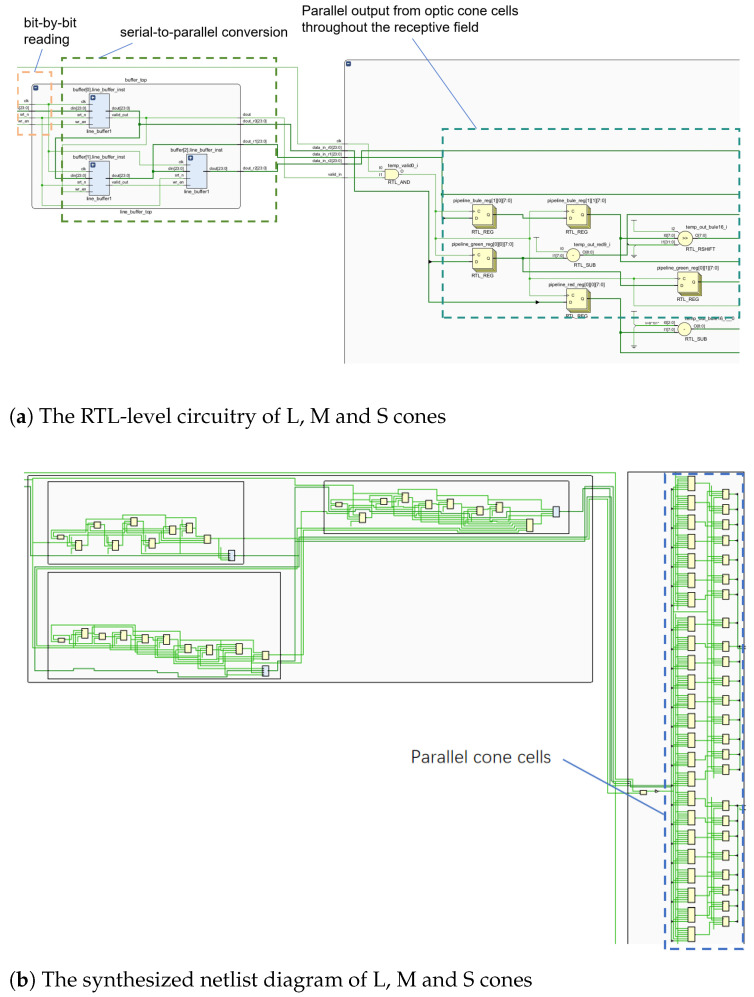
Circuit diagram for L, M and S cone cells within the receptive field.

The register pipeline stores the entire receptive field, reading it simultaneously after filling. The R, G and B information corresponds to L, M and S cone cells. Figure 11 shows the FPGA implementation from image reading to L, M and S cone extraction. Figure 11 exhibits the RTL-level circuit, while Figure 11b depicts the synthesized netlist. Each 8-LUT memory circuit represents a cone cell. These cone cells are fed simultaneously to the sensory field. The RTL circuit’s buffer represents the pipeline cache line. Right-side modules in Figure 11a represent L, M and S cone RTL circuitry. The RTL-level circuit (Figure 11a), and register pipeline netlist (Figure 11b) are shown below.

### 4.2. FPGA Implementation of Neuronal Opponent Computation

#### 4.2.1. Neuronal Opponent Computational Primitivization of K, M and P Pathways

**Figure 12:**
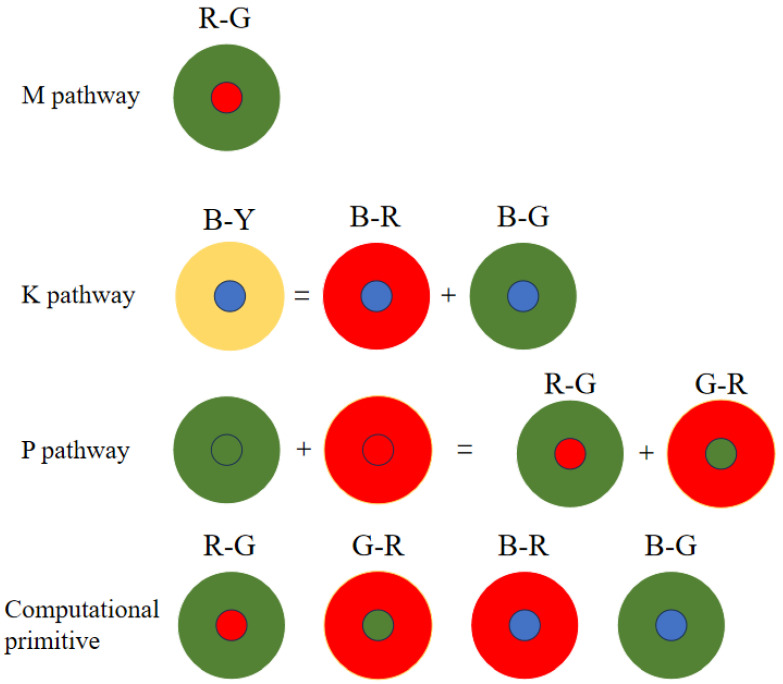
Opponent computational model and computational primitives.

The K, P and M channels can be independently processed. While sharing some photoreceptor cells, electronic image pixels contain R, G and B information corresponding to L, M and S cones. This act allows for identical receptive field sizes, optimizing LUT usage. Each receptive field computes all three pathways (Figure 12).

Given the center-surround organization of ganglion cells, the K, M and P pathways exhibit antagonistic behavior (Figure 12). To optimize resource utilization, four computational primitives (R-G, B-G, B-R and G-R) are introduced (Figure 12), leveraging the inherent symmetry of weight matrices for compression.

Similar opponent computations allow a single primitive opponent circuit to handle three channels per receptive field. In the blue-yellow opponent, the optic cone calculation for the output is $C_{B-Y}$:

(7) $$C_{B-Y}=S_{cen}-\left(M_{per}+L_{per}\right)/2$$

Using the law of conjunction, this can be further reduced to:

(8) $$C_{B-Y}=\left(S_{cen}-M_{per}\right)/2+\left(S_{cen}+L_{per}\right)/2$$

Consequently, both B-R and B-G opponents can be computed simultaneously, followed by signal superposition. The homochromatic opponent of the P pathway is calculated as shown, with $C_{R+G}$ representing the M pathway opponent output:

(9) $$C_{R+G}=\left(L_{cen}-L_{per}\right)/2+\left(M_{cen}-M_{per}\right)/2$$

which can be further reduced to:

(10) $$C_{R+G}=\left(L_{cen}-M_{per}\right)/2+\left(M_{cen}-L_{per}\right)/2$$

where $L_{cen}$ and $L_{per}$ represent the red center and red periphery, respectively; $M_{cen}$ and $M_{per}$ signify the green center and green periphery, respectively. The M pathway’s bicolor opponent uses the same cones as the P pathway. For resource optimization, a unified computational model for M and P pathways is proposed, minimizing resource usage while efficiently processing visual signals.

(11) $$C_{R-G}=\left(L_{cen}-M_{per}\right)/2$$

**Figure 13:**
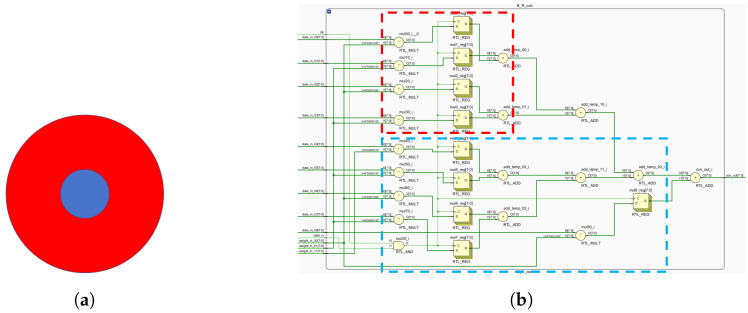
B-R opponent motifs and circuit layout. (**a**) B-R computing primitives. (**b**) The circuit layout corresponding to B-R, where red indicates the receptive field surround and blue indicates the receptive field center.

**Figure 14:**
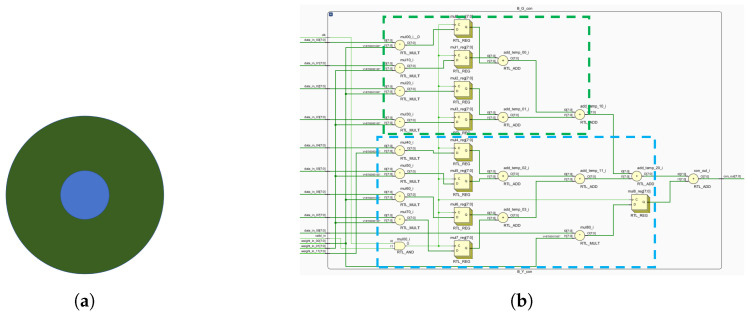
B-G opponent motifs and circuit layout. (**a**) B-G computing primitives. (**b**) The circuit layout corresponding to B-G, where green indicates the receptive field surround and blue indicates the receptive field center.

**Figure 15:**
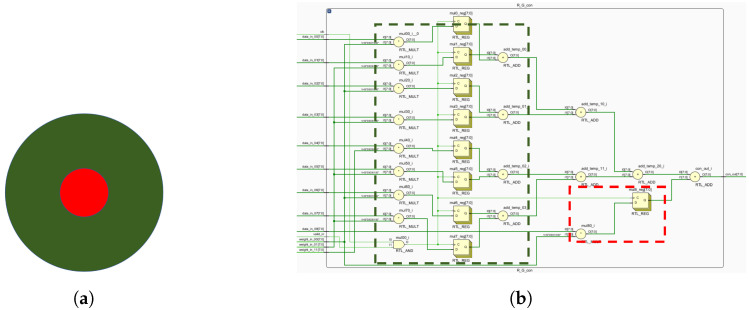
R-G opponent motifs and circuit layout. (**a**) R-G computing primitives. (**b**) The circuit layout corresponding to R-G, where green indicates the receptive field surround and red indicates the receptive field center.

**Figure 16:**
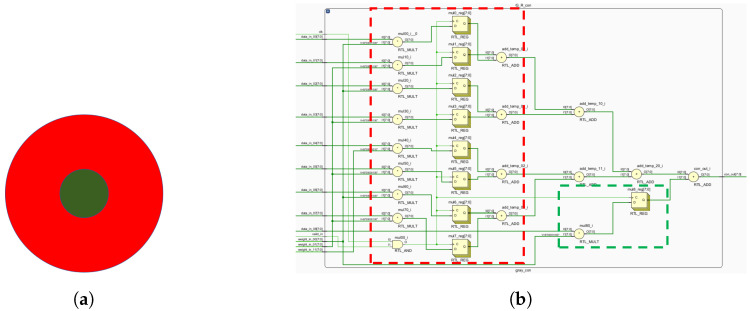
B-G opponent motifs and circuit layout. (**a**) G-R computing primitives. (**b**) The circuit layout corresponding to G-R, where red indicates the receptive field surround and green indicates the receptive field center.

Figure 13 shows computations for both central and peripheral cones. These mappings enable FPGA-based ganglion cell implementation (Figure 14, Figure 15 and Figure 16).

#### 4.2.2. Circuit Modeling of Single Receptive Field K, M and P Pathway Opponent Calculations

**Figure 17:**
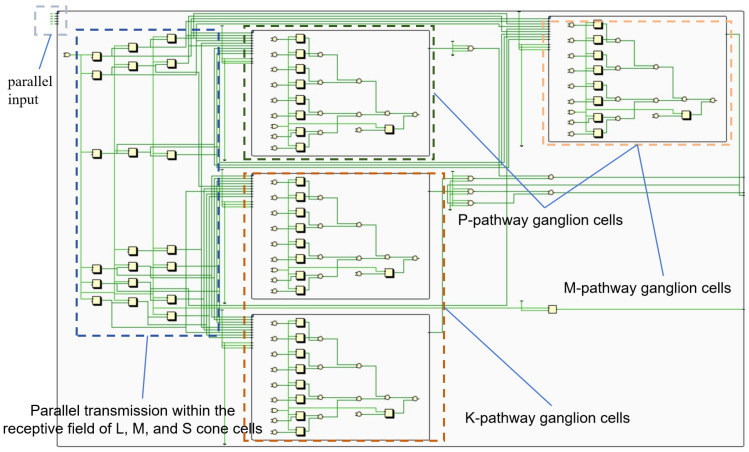
RTL circuit diagram for single receptive field opponent computational model.

Figure 17 shows a receptive field model, with components representing K, M and P pathways. The register group represents L, M and S optic cone cells, reflecting parallel photoreceptor transmission. Colored boxes signify the computational primitives for K, M and P pathways. The P-pathway uses both R-G and G-R computations, while the M-pathway uses R-G. To optimize resources, the R-G module’s output is multiplexed with the M-channel. Analyzing the resource and power usage of a single receptive field estimates the maximum number of parallel receptive fields on the FPGA.

#### 4.2.3. Opponent Computational Multiplication Tree Design

**Figure 18:**
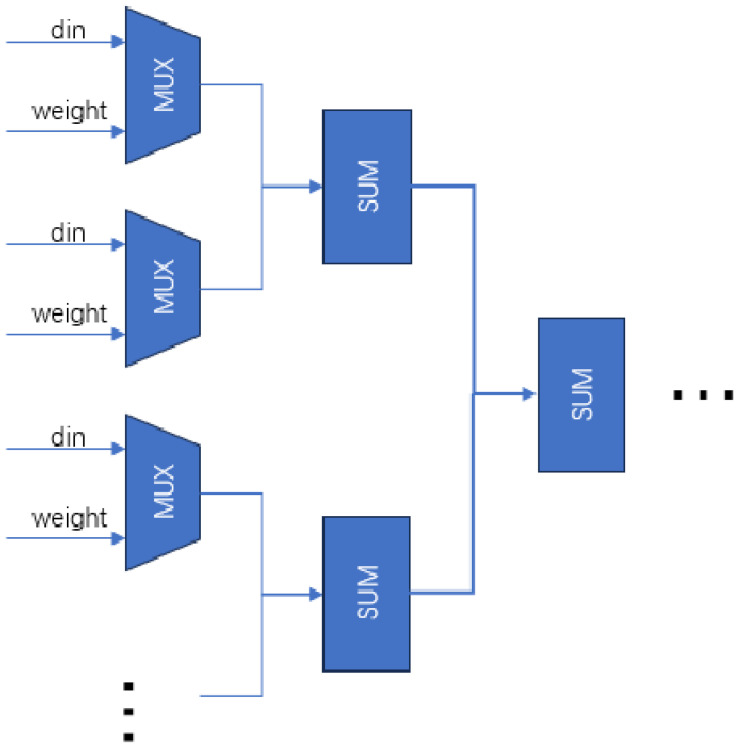
Parallel computation model for vector multiplication.

Opponent computations, such as the multiply–accumulate operations in neural networks, subtract peripheral signal strengths from central ones. CPUs perform these operations sequentially, but FPGAs use parallel processing for efficient convolutional computations. By dividing vector multiplication into parallel multiplication and addition trees, FPGAs achieve intercellular nucleus level parallelism, optimizing computation efficiency (Figure 18).

Parallel multiplication requires simultaneous access to receptive field data. The preceding module stores L, M and S cone cell data in a register pipeline array cache for parallel readout. This paper optimizes parallel multiplication and addition, reducing the critical path from 10 to 5 compared to sequential computation.

**Figure 19:**
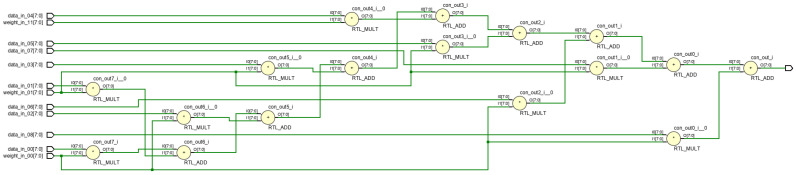
Optimizing pre-vector multiplication order for circuit path computation.

**Figure 20:**
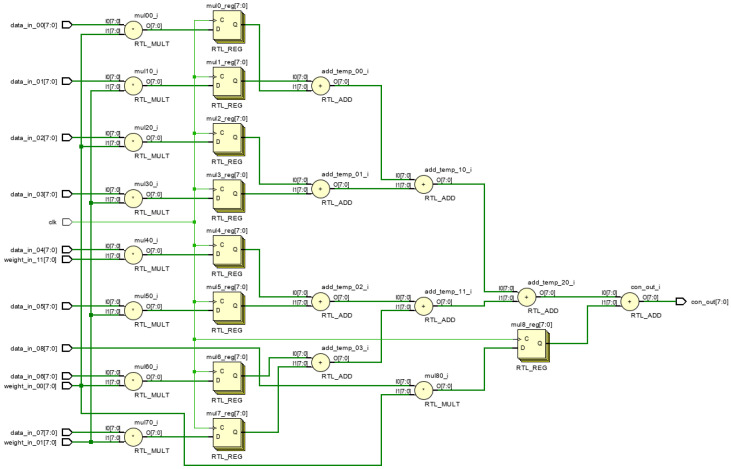
RTL circuitry for optimized parallel vector multiplication model.

Weight matrix symmetry allows matrix storage, using upper triangular matrices to store three weights for a 3 × 3 receptive field. Figure 19 and Figure 20 illustrates the RTL circuit diagram for a 9-dimensional parallel vector product module, demonstrating critical path reduction.

## 5. Parallelism and Resource Utilization Analysis

### 5.1. Individual Receptive Field Resource Analysis

To simulate the visual system’s parallel neurons, understanding FPGA resource requirements is crucial. This paper uses the neuron’s receptive field to estimate the FPGA resources and the maximum number of neurons that can be transmitted simultaneously. For the Artix-7 family of xc7a200tsbv484-1 FPGAs, Table 2, Table 3 and Table 4 summarize the resource usage. They cover 3 × 3 and 5 × 5 receptive fields. These tables provide insights into the FPGA resource requirements for the neuronal model. The optimized 3 × 3 receptive field uses far fewer resources than the pre-optimized one. This shows that resource consumption can be effectively reduced by the model in this study. The 5 × 5 receptive field uses almost three times more resources than the 3 × 3 receptive field. This is because as the receptive field becomes larger, the nodes of the synapse become more numerous, requiring more resources to be consumed. The experimental results are consistent with the biological conclusions.

The computation module primarily uses look-up tables (LUTs), which consumes a significant portion of the FPGA’s resources. Consequently, the maximum number of neurons that can be processed in parallel on an FPGA board depends largely on the availability of LUTs. Notably, multiplication is performed via shift operation in the vector product tree, without using multiplier resources such as DSP.

Comparing resource usage between 5 × 5 and 3 × 3 receptive fields, the former exhibits greater parallelism. Both pipelined and parallel computations use more resources for the 5 × 5 receptive fields. However, parallel computation requires fewer resources due to the optimization of part of the multiplication in this paper.

**Figure 21:**
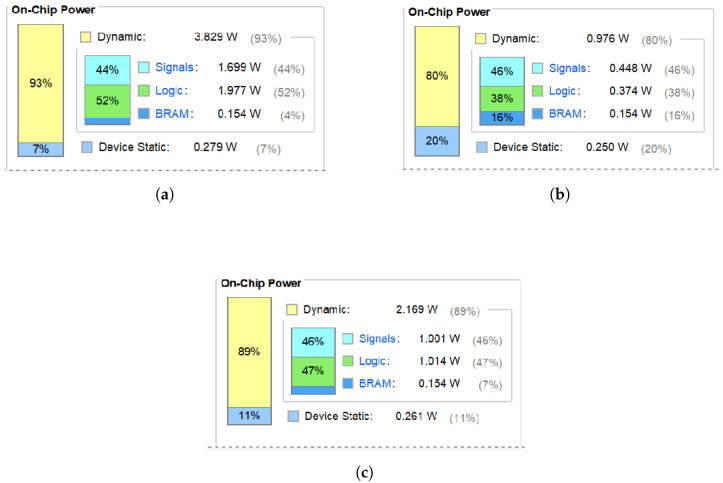
Power consumption of two receptive fields at 100 MHz. (**a**) Power summary for a 3 × 3 receptive field before optimization. (**b**) Power summary for an optimized 3 × 3 receptive field. (**c**) Power summary for an optimized 5 × 5 receptive field.

Figure 21a depicts the power consumption of a 3 × 3 receptive field, while Figure 21c highlights that of an optimized 5 × 5 receptive field. Before optimization, the 3 × 3 field consumes 4.108 W (1.977 W for logic). After optimization, the 3 × 3 field consumes 1.23 W and the 5 × 5 field consumes 2.43 W (1.014 W for logic). Despite the larger receptive field, the optimized model significantly reduces power consumption. The logic uses half the power of the original. As shown in Figure 21a,b, the power required to compute a receptive field is much smaller after the optimization. From Figure 21a,c, it can be seen that the larger the size of the receptive field, the higher the power.

**Figure 22:**
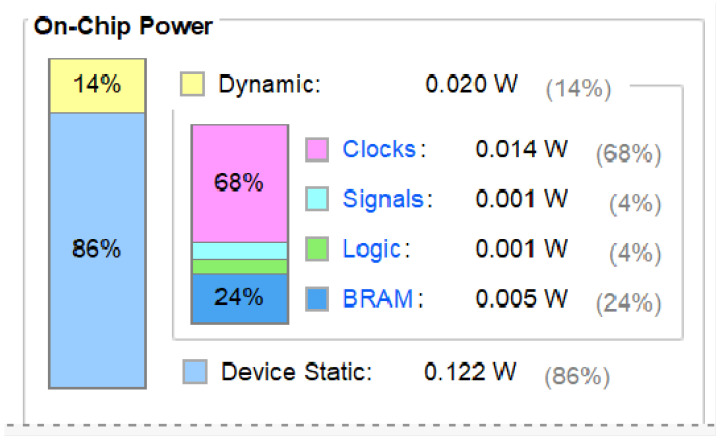
Power usage of a 5 × 5 receptive field at 200 MHz.

The Artix-7 xc7a200tsbv484-1 model can reach speeds up to 200 MHz with a power consumption of just 0.142 W (Figure 22). As shown in Figure 21c and Figure 22, higher frequencies result in lower power. Each receptive field uses 0.142 W. Power consumption increases significantly when thousands of parallel fields are used. Considering the human eye has millions of optic cone cells, it is impractical for a single visual system to consume thousands of Watts. Consequently, exploring energy-saving design principles inspired by biological systems is crucial.

### 5.2. Vector Product Multiplication Tree Resource Analysis and Optimization

The vector product computation uses only LUTs (Table 4), avoiding DSP which needs 4 cycles per calculation. Weights are simplified with Taylor Expansion during initialization, resulting in 310 LUTs for a 9-dimensional field. Expanding to 5 × 5 fields increases LUT usage to 1132 due to passing the weights matrix and using register matrices.

To emulate the integrated storage and computation seen in biological systems, weights can be written during initialization, and addition can follow multiplication directly. This optimization reduces LUT usage to 334, a decrease of 70.5 compared to the pre-optimization stage. Table 4 Resource analysis before and after single sensory field optimization.

### 5.3. Computational Primitive Effectiveness Analysis

In the 3 × 3 sensory field (Table 1), implementing the neuronal three-way pathway uses 1397 LUTs, mainly for parallel computation involving four computational primitives.

B_G and B_R form the K-pathway, R_G represents the M-pathway model and R_G plus G_R jointly make up the P-pathway model. The P-pathway model includes the M-pathway computation, allowing direct use of M-pathway output from R_G.

Table 1 shows that one R_G module uses 313 LUTs. By computational primitives, one R_G module is saved, reducing resource use by 18% of the total 1710 LUTs. Similarly, in a 5 × 5 field, computational primitives save one R_G model, resulting in about 16% savings year-on-year. Consequently, using computational primitives instead of full opponent computation cuts resource consumption by roughly one-sixth.

### 5.4. Parallel Receptive Field Analysis

**Figure 23:**
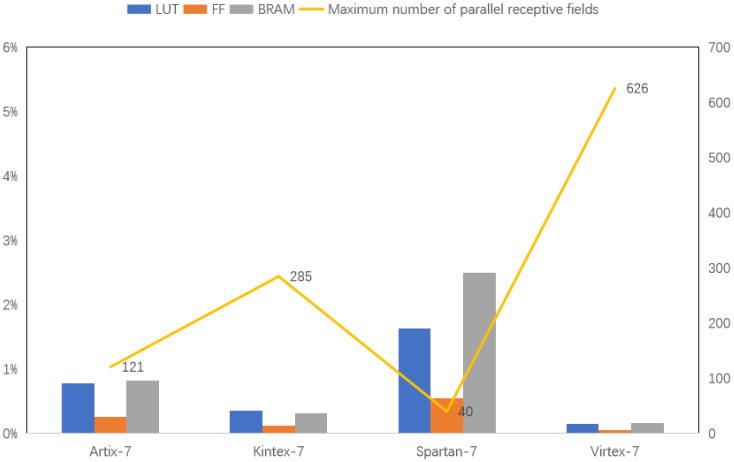
Resource allocation and maximum receptive fields supported in FPGA models.

Figure 23 compares resource usage for a single receptive field across Xilinx 7 series FPGAs: Aritix-7, Kintex-7, Spartan-7 and Virtex-7 (100 MHz). The experiments use models with the most FF, BRAM and LUT resources. Among these four types, the FPGA version chosen for the experiments, with the most resources, are xc7a200tsbv484-1, xc7k480tiffv1156-2L, xc7s100fgga676-1Q and xc7vx1140tflg1930-1, respectively. The bar graph shows resource allocation for each FPGA, while the line graph indicates the theoretical maximum number of parallel receptive fields. The data reveals that FPGA resources significantly limit the number of receptive fields that can be processed simultaneously.

Even with resource-rich FPGA boards, such as the Virtex-7 model, processing more than 630 receptive fields in parallel is impractical. In contrast, the human visual system has millions of optic cones and even more ganglion cells. This highlights the complexity and efficiency of the biological system. Modern technology has not yet matched this level of sophistication. Biological systems achieve clear vision with low energy through highly parallel processing, a challenge still unmet by current technology. As a result, other bionics must continue exploring ways to achieve similar efficiency and energy conservation.

### 5.5. Comparison with Other Works

We put the experimental models on CPU and GPU for the experiment. Under the same conditions of receptive field and parallelism set to 600, the experimental results are shown in Table 5. In throughput metrics, we’re 3150 times better than the CPU. And power consumption is very favorable. Compared to GPUs, the higher throughput also requires greater power consumption. And GPUs require more space, which is not in line with the purpose of brain-like design in small and light devices.

Table 6 shows that our model is able to simulate biologically neurons better, the most neurons in the first row is due to the use of 5 FPGAs. it can be seen from the table that the CUPS (computations per second) of our model is also relatively high. This is attributed to our vector product computation, finer-grained design and higher number of parallel neurons. It also shows that fine-grained biological models can be realized with biologically based features.

### 5.6. Experimental Results

**Figure 24:**
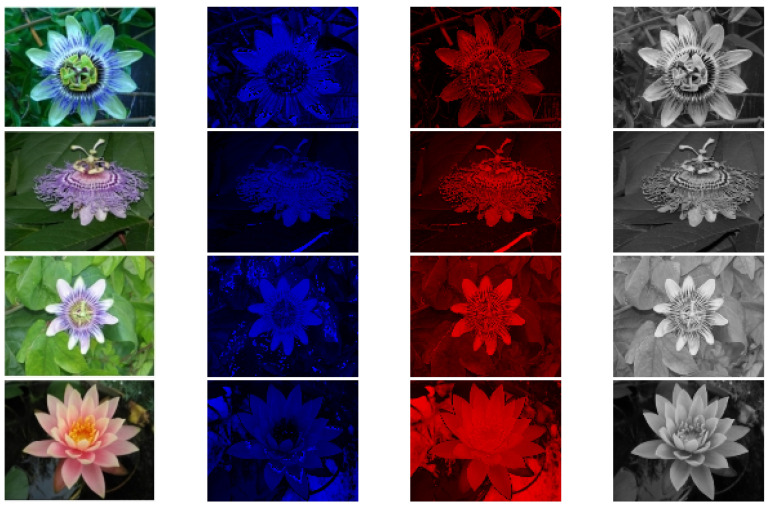
Experimental results for K, M and P pathways.

**Figure 25:**
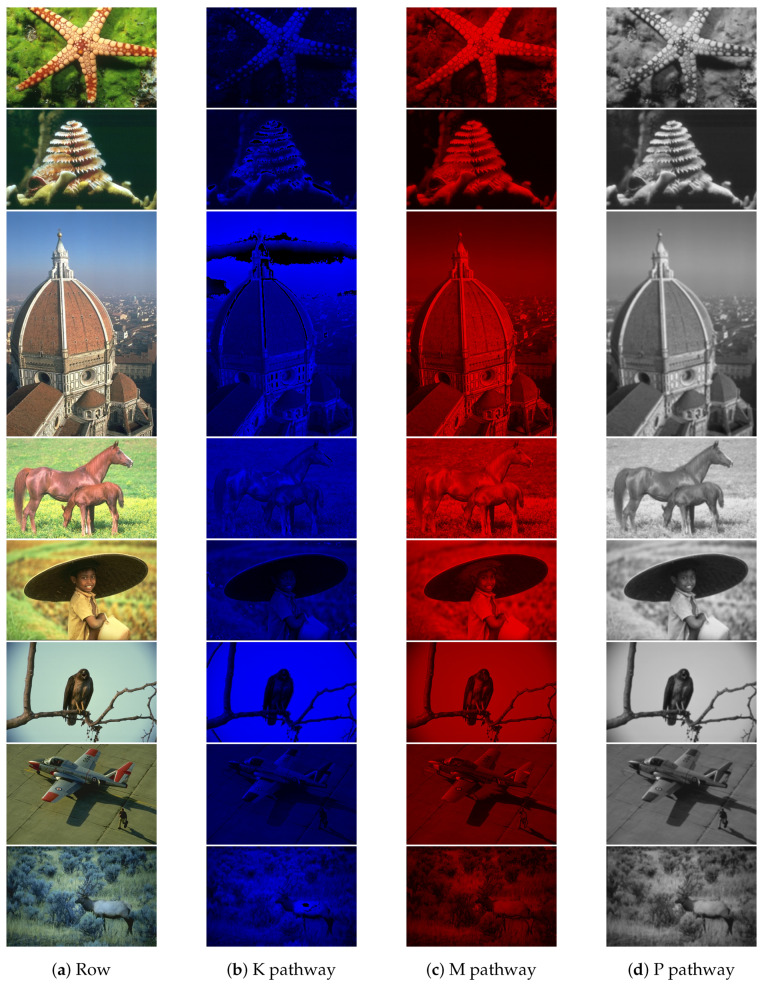
Experimental results for K, M and P pathways. The data source for this part of the experiment is the BSD dataset [31].

Figure 24 shows the experimental results from this study using a dataset from the University of Oxford [30]. The data in Figure 25 are derived from the BSD dataset [31]. The table has four columns. The first column displays the original images. The second column shows the blue-yellow antagonized K pathway, mostly outside the optic recess. The third column depicts the red-green M pathway, located only in the central recess. The fourth column features the P pathway, positioned outside the optic recess, with homochromatic opponent processing. The figure highlights the distinct characteristics of each visual pathway.The P pathway processes wide visual stimuli. It often produces slightly blurred images. The K and M pathways work together to provide finer detail and color representation.

These three pathways represent separate channels that transmit information in parallel in living things, and visual information located in different regions of the retina. This information is transmitted to the LGN before it is further processed. Many studies have completely ignored this point, and if the information transmitted at the lower levels is completely different from the biological model, then the final model is certainly not an accurate biological model.

This analysis highlights how the retina separates visual information into different channels. These channels are then processed by the higher visual cortex. This demonstrates a hierarchical approach to visual processing. While not the most efficient, the biological visual system is one of the most comprehensive one, making further research valuable for understanding visual processing.

### 5.7. Validation of Bionic Results

**Figure 26:**
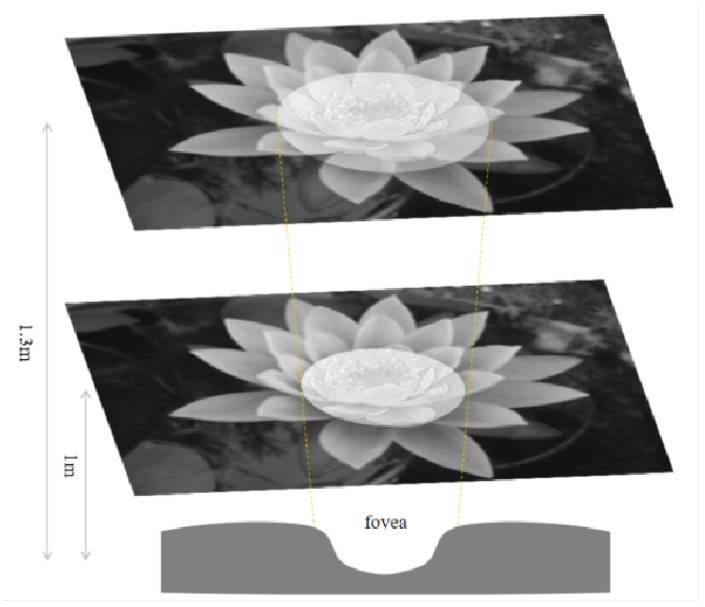
Mapping range of the fovea at various distances.

The central concavity of the human eye processes visual information within about 5° [32]. Figure 26 shows the information captured by the M P pathways. The M pathway focuses on the central area, while the P pathway extends beyond it. For example at 1 m, the central concavity perceives a 43.7 mm circle (1000×tan(5/2°). At 1.3 m, it expands to 56.8 mm (Figure 26). Many studies focus directly on higher-level visual processing systems and model their functions in a logical approximation. However, image information in biological vision is transmitted from lower to higher levels. The retinal pathways that transmit to the outer LGN are K, M and P, which means that the image information transmitted from the LGN to the primary cortex should be as shown in Figure 26. The images generated from different parts of the retina located in different parts of the retina are inconsistent, rather than a whole sheet of images processed with a single model. A proper bionic model is only possible if the visual information transfer process of living beings is completely imitated in the underlying layer.

## 6. Conclusions and Discussion

Biological-vision processes light signals simultaneously through the retina and LGN before reaching the cortex. While neuromorphic computing (NC) focuses on the cerebral cortex, image information is pre-processed in the early visual stages. This paper models the primate retina’s parallel pathways for NC. Biological vision excels at parallel processing, a feat challenging for traditional computers. Due to the inadequacy of VN architecture, FPGAs offer a potential solution by modeling the primate retina’s parallel pathways. Our model independently processes the K, M and P channels for color perception, fine details and peripheral blurring, respectively. Horizontal parallelism is achieved through receptive fields, mimicking biological computation. Experimental results indicate that a single receptive field consumes nearly 0.14 W with resource-rich FPGA boards computing only 626 fields simultaneously, highlighting the need for enhanced parallelism. Additionally, this paper identifies two areas for further exploration: increasing parallelism and extending the model. Current FPGA capabilities allow for only 626 parallel receptive fields, which is significantly less than found in biological systems. The model covers only the retina to the LGN, extending it to the cerebral cortex could better replicate the entire visual system. Future research should focus on integrating higher-level visual cortices for greater efficiency and parallelism.

## Figures and Tables

**Table 1:** K, M and P channels: A comparative analysis.

	P-Pathway	M-Pathway	K-Pathway
Associated regions in the retina	Outside of the fovea	Inside of the fovea	Interior and Exterior of the fovea
Layers of ganglion cell projections to the LGN	magnocellular layers of the LGN	parvocellular layers of the LGN	intercalated layers of the LGN
Optic cones involved in opponent	L and M	L and M	S, M and L
Opponent type	Homochromatic opponent	Two-color opponent	Two-color opponent
Opponent calculation	$L_{cen}-L_{per}$ $M_{cen}-M_{per}$	$L_{cen}-M_{per}$	$S_{cen}-\left(M_{per}+L_{per}\right)/2$
functionality	Low-precision visual information outside the fovea	Higt-precision visual information inside the fovea	Color Space Feeling

**Table 2:** Computational resources required for a 3 × 3 receptive fields.

Resources	Slice LUTs (303,600)	Slice Registers (607,200)	Slice (75,900)	BRAM (1030)
Project	1397 (4.6‰)	466 (7.7‰)	547 (7.2‰)	2(1.9‰)
B_G_con Unit	313	51	108	0
B_R_con Unit	309	49	110	0
G_R_con Unit	316	51	123	0
R_G_con Unit	316	51	132	0

**Table 3:** Computational resources needed for an optimized 3 × 3 receptive fields.

Resources	Slice LUTs (303,600)	Slice Registers (607,200)	Slice (75,900)	BRAM (1030)
Projecct	410 (1.4‰)	302 (0.5‰)	158 (2.1‰)	2 (1.9‰)
B_G_con Unit	86	0	27	0
B_R_con Unit	64	0	29	0
G_R_con Unit	68	0	27	0
R_G_con Unit	68	0	27	0

**Table 4:** Computational resources needed for an optimized 5 × 5 receptive fields.

Resources	Slice LUTs (303,600)	Slice Registers (607,200)	Slice (75,900)	BRAM (1030)
Projecct	1047 (3.4‰)	700 (1.2‰)	370(4.8‰)	3 (2.9‰)
B_G_con Unit	204	0	78	0
B_R_con Unit	191	0	72	0
G_R_con Unit	206	0	72	0
R_G_con Unit	202	0	73	0

**Table 5:** Comparison results with CPU adn GPU.

Platform	Throughput (k)	Power (W)
CPU(AMD 4800H )	60	46
GPU(RTX 3060Ti)	335,432	451
FPGA(xc7a200tsbv484-1)	195,312	8

**Table 6:** Comparison of CUPS Performance.

	Number of Neurons	CUPS (M)
[27]	66	792
[28]	48 × 10^6^	199
[29]	49	300
[1]	38	6463
Ours	600	11,444

## Data Availability

No new data were created.

## References

[1] Wei H., Ye J., Li J., Wang Y. (2023). Design and Simulation of a Hierarchical Parallel Distributed Processing Model for Orientation Selection Based on Primary Visual Cortex. Biomimetics.

[2] Roy K., Jaiswal A., Panda P. (2019). Towards spike-based machine intelligence with neuromorphic computing. Nature.

[3] Deng L., Wang G., Li G., Li S., Liang L., Zhu M., Wu Y., Yang Z., Zou Z., Pei J. (2020). Tianjic: A unified and scalable chip bridging spike-based and continuous neural computation. IEEE J. Solid-State Circuits.

[4] Dahasert N., Öztürk İ., Kiliç R. (2012). Experimental realizations of the HR neuron model with programmable hardware and synchronization applications. Nonlinear Dyn..

[5] Korkmaz N., Öztürk İ., Kılıç R. (2016). The investigation of chemical coupling in a HR neuron model with reconfigurable implementations. Nonlinear Dyn..

[6] Nazari S., Faez K., Amiri M., Karami E. (2015). A digital implementation of neuron–astrocyte interaction for neuromorphic applications. Neural Netw..

[7] Yang S., Wang J., Li S., Li H., Wei X., Yu H., Deng B. (2016). Digital implementations of thalamocortical neuron models and its application in thalamocortical control using FPGA for Parkinson’s disease. Neurocomputing.

[8] Yang S., Wang J., Li S., Deng B., Wei X., Yu H., Li H. (2015). Cost-efficient FPGA implementation of basal ganglia and their Parkinsonian analysis. Neural Netw..

[9] Soleimani H., Bavandpour M., Ahmadi A., Abbott D. (2014). Digital implementation of a biological astrocyte model and its application. IEEE Trans. Neural Netw. Learn. Syst..

[10] Krizhevsky A., Sutskever I., Hinton G.E. (2012). Imagenet classification with deep convolutional neural networks. Advances in Neural Information Processing Systems.

[11] Wässle H. (2004). Parallel processing in the mammalian retina. Nat. Rev. Neurosci..

[12] Mahowald M. (1994). An Analog VLSI System for Stereoscopic Vision.

[13] Zaghloul K.A., Boahen K. (2004). Optic nerve signals in a neuromorphic chip I: Outer and inner retina models. IEEE Trans. Biomed. Eng..

[14] Keener J., Sneyd J. (2009). Mathematical Physiology: II: Systems Physiology.

[15] Williams D.S. (2008). Usher syndrome: Animal models, retinal function of Usher proteins, and prospects for gene therapy. Vis. Res..

[16] Hartong D.T., Berson E.L., Dryja T.P. (2006). Retinitis pigmentosa. Lancet.

[17] Wong B. (2011). Color blindness. Nat. Methods.

[18] Dacey D.M. (2000). Parallel pathways for spectral coding in primate retina. Annu. Rev. Neurosci..

[19] Ghanbarpour M., Haghiri S., Hazzazi F., Assaad M., Chaudhary M.A., Ahmadi A. (2023). Investigation on Vision System: Digital FPGA Implementation in Case of Retina Rod Cells. IEEE Trans. Biomed. Circuits Syst..

[20] Ghanbarpour M., Naderi A., Haghiri S., Ahmadi A. (2021). An efficient digital realization of retinal light adaptation in cone photoreceptors. IEEE Trans. Circuits Syst. I Regul. Pap..

[21] Voroshazi Z., Nagy Z., Szolgay P. An advanced real-time, multi-channel emulated-digital retina model implementation on FPGA. Proceedings of the 2008 11th International Workshop on Cellular Neural Networks and Their Applications.

[22] Voroshazi Z., Nagy Z., Szolgay P. (2009). FPGA-based real time, multichannel emulated-digital retina model implementation. EURASIP J. Adv. Signal Process..

[23] Kim I.J., Zhang Y., Yamagata M., Meister M., Sanes J.R. (2008). Molecular identification of a retinal cell type that responds to upward motion. Nature.

[24] Solomon S.G., Lennie P. (2007). The machinery of colour vision. Nat. Rev. Neurosci..

[25] Rodieck R.W. (1965). Quantitative analysis of cat retinal ganglion cell response to visual stimuli. Vis. Res..

[26] Schwartz J.H. (2000). Principles of Neural Science.

[27] Deng B., Fan Y., Wang J., Yang S. (2021). Reconstruction of a Fully Paralleled Auditory Spiking Neural Network and FPGA Implementation. IEEE Trans. Biomed. Circuits Syst..

[28] Glackin B., Harkin J., McGinnity T.M., Maguire L.P., Wu Q. Emulating spiking neural networks for edge detection on FPGA hardware. Proceedings of the 2009 International Conference on Field Programmable Logic and Applications (FPL).

[29] Długosz R., Kolasa M., Szulc M. An FPGA implementation of the asynchronous programmable neighborhood mechanism for WTM Self-Organizing Map. Proceedings of the 18th International Conference Mixed Design of Integrated Circuits and Systems—MIXDES 2011.

[30] 102 Category Flower Dataset. http://www.robots.ox.ac.uk/~vgg/data/flowers/102/index.html.

[31] Martin D., Fowlkes C., Tal D., Malik J. A database of human segmented natural images and its application to evaluating segmentation algorithms and measuring ecological statistics. Proceedings of the Eighth IEEE International Conference on Computer Vision (ICCV).

[32] Kolb H., Nelson R.F., Ahnelt P.K. (1995). The Architecture of the Human Fovea. Webvision: The Organization of the Retina and Visual System [Internet].

